# Multiple Cutaneous Adverse Reactions Secondary to Nivolumab Therapy in an Elderly Male

**DOI:** 10.7759/cureus.81542

**Published:** 2025-03-31

**Authors:** Divya Bhatia, Ekta Gupta, Anupama Bains, Deepak Vedant

**Affiliations:** 1 Dermatology, All India Institute of Medical Sciences, Jodhpur, IND; 2 Pathology and Lab Medicine, All India Institute of Medical Sciences, Jodhpur, IND

**Keywords:** generalized eczema, immune checkpoint inhibitor, localized vitiligo, malignant melanoma metastasis, nivolumab-related adverse events, sweet's syndrome

## Abstract

Nivolumab is a human IgG4, PD-1 inhibitor that is approved for the treatment of advanced melanoma. We hereby report a patient of metastatic melanoma who developed multiple cutaneous side effects, including Sweet's syndrome, lichenoid eruption, and vitiligo-like depigmentation after initiation of the nivolumab therapy. He first started developing vitiligo-like depigmentation over the lips, which appeared after five months. Later, he developed a mildly itchy lichenoid eruption over the thighs. The most recent and disabling one was the appearance of erythematous edematous painful plaques studded with pustules and vesicles with fever. The latter was diagnosed as Sweet's syndrome. Nivolumab was stopped, and the patient was started on oral dapsone 100 mg per day with topical mometasone 0.1% cream once a day application for these lesions. The patient had complete resolution of edematous plaques after four weeks of follow-up. Also, the subsequent dose of nivolumab was decreased by 20%, and this was not followed by the recurrence of Sweet's syndrome; however, vitiligo-like depigmentation persisted.

## Introduction

The advent of therapies like immune checkpoint inhibitors (ICI) has become the cornerstone of the management of many solid organ malignancies in the recent past. However, the immune blockade caused by these agents can lead to immunological intolerance and cause immune-related adverse events, including cutaneous lesions. Programmed cell death 1 (PD-1) is a key immune checkpoint receptor that is expressed by activated T cells and functions primarily in peripheral tissues. Nivolumab is a monoclonal human IgG4 antibody against PD-1 that has been approved for the management of advanced melanoma. Though these emerging therapies are effective in the management of malignancies, they are not free of side effects. Common immune-mediated cutaneous events identified in patients receiving PD-1 inhibitors include lichenoid reactions, eczema, vitiligo, and pruritus [1]. The frequency of the cutaneous adverse events appears to increase with increasing exposure to the treatment, thus requiring long-term monitoring for patients who are receiving PD-1 therapy [1]. The time of onset of the cutaneous events varies, ranging from four to 10 months, and occurs later than that seen with the CTLA-4 inhibitors. The cutaneous and mucocutaneous adverse events associated with PD-1 inhibitors are generally self-limited and readily manageable with topical steroids alone. We hereby present a case of malignant melanoma that received nivolumab therapy and developed Sweet’s syndrome, vitiligo-like depigmentation, and eczematous eruption at various time points during the course of the nivolumab therapy.

## Case presentation

**Figure 1:**
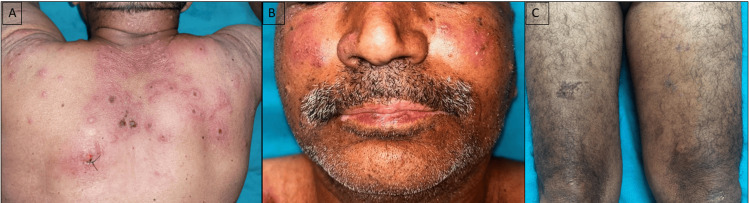
Various morphological clinical presentation in index patient. (A) Multiple well-defined urticarial edematous plaques with central pseudovesiculation, pustules, and crusting, (B) depigmented macules over the lips with residual rim of pigmentation around the lip margins, (C) well-defined erythematous plaque with scaling over the upper thigh.

**Figure 2:**
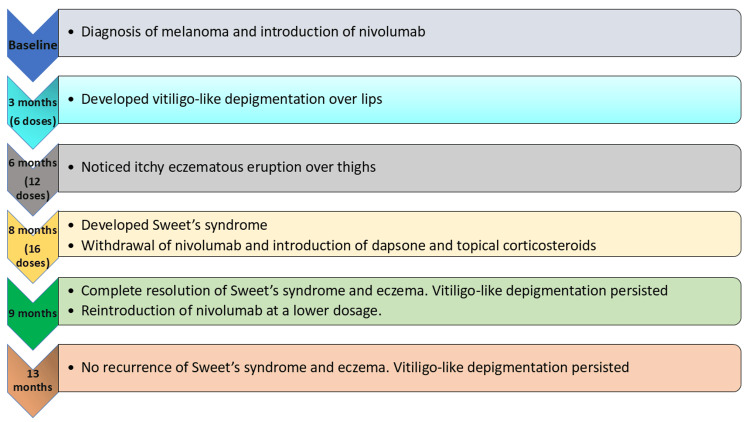
Timeline chart: onset and course of various skin manifestations after nivolumab therapy. The image is created by the author (Divya Bhatia) of this study.

An elderly patient was diagnosed with malignant melanoma of the left foot with metastasis in the left inguinal lymph node over the past eight months. He was initially started on anti-PD-1 therapy, i.e., nivolumab 240 mg every fortnightly. He first developed depigmentation of both lips, which was gradually progressive in nature, followed by eruption of itchy eczematous plaques over both thighs, and lastly, he also developed painful erythematous lesions over the head and neck, arms, and upper back for the past three days. These lesions developed after three months (six cycles), six months (12 cycles ), and eight months (16 cycles) of nivolumab therapy. He received his last dose one week prior to the onset of his recent skin eruption. These lesions were associated with pustulation and intermittent pus discharge. As well as it was also associated with low-grade fever. There was no history of joint pain or any other systemic complaints. Examination revealed multiple erythematous edematous tender plaques with central pseudo-vesiculation, crusting, and pustules over the face, upper back, and bilateral upper arms (Figure 1A). In addition, multiple lichenoid scaly plaques were present over the thighs (Figure 1B). There was depigmentation over both upper and lower lips along the margins of the lips (Figure 1C). There were no lesions noted inside the oral mucosa. The onset of various cutaneous lesions after nivolumab introduction is explained in Figure 2.

**Figure 3:**
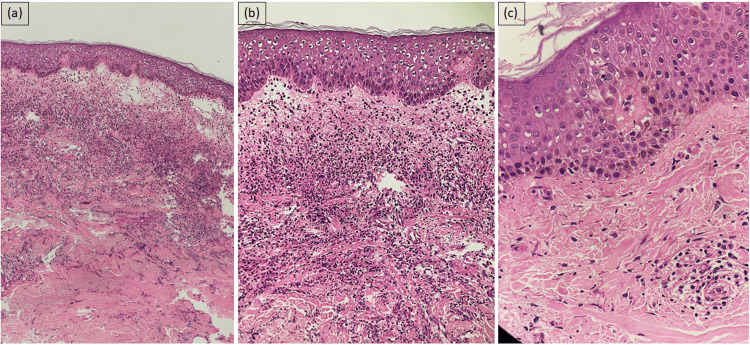
Histopathology from the edematous tender plaques. (A) Low-power view of biopsy from edematous urticarial plaque over upper back shows basket weave hyperkeratosis and flattening of rete ridges in the epidermis. There is a dense collection of inflammatory infiltrates in the upper dermis along with edema (H&E, 10x). (B) High-power view of biopsy from edematous urticarial plaque over upper back shows basket weave hyperkeratosis and flattening of rete ridges in the epidermis. Dermis shows a dense collection of acute inflammatory infiltrate, predominantly of neutrophils, along with edema (H&E, 20x). (C) Histopathology of thigh lesion showed hyperkeratosis, spongiosis, flattened rete ridges, and a moderate inflammatory infiltrate of lymphocytes, plasma cells, and few neutrophils in the upper dermis (H&E, 40x).

Due to temporal correlation with drug initiation, we considered the possibility of nivolumab-induced Sweet's syndrome, vitiligo, and eczematous eruption. Blood workup revealed a total leukocyte count of 10,980/mm^3^ with 71% neutrophils. A complete workup for fever evaluation was done, including blood culture and sensitivity, erythrocyte sedimentation rate (ESR), C-reactive protein (CRP), urine culture and sensitivity, and dengue NS1 antigen and malaria parasite card test, which did not reveal any significant abnormality except raised CRP and ESR. Also, workup for tropical infections like scrub, salmonella, and leptospira was negative. Histopathology of tender plaques revealed dermal edema and dense inflammatory infiltrate in the upper dermis, comprised of predominantly neutrophils (Figures 3A, 3B). Histopathology of thigh lesion showed hyperkeratosis, spongiosis, and flattened rete ridges and a moderate inflammatory infiltrate of lymphocytes, plasma cells, and few neutrophils in the upper dermis (Figure 3C).

**Figure 4:**
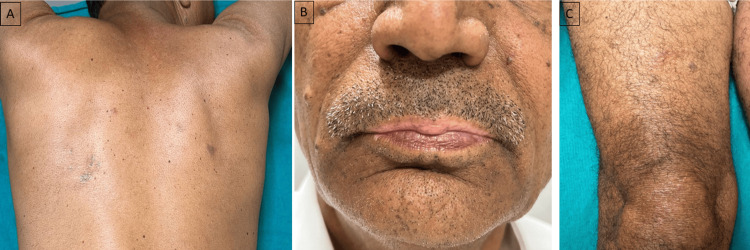
Post-treatment images. Post-treatment images of the patient show near complete improvement of Sweet's syndrome lesions (A), there was improvement in vitiligo-like depigmentation but it persisted (B), and there was significant improvement in thigh eczematous eruption (C).

Nivolumab was discontinued, and the patient was started on oral dapsone 100 mg per day along with topical mometasone furoate 0.1% cream once a day application. Patient reported complete resolution of Sweet’s syndrome and eczematous eruption at four weeks. Also, the subsequent dose of nivolumab was decreased by 20% and further continued for four months. It was not followed by recurrence of the Sweet’s syndrome and eczema; however, vitiligo-like depigmentation persisted (Figures 4A-4C). For vitiligo-like depigmentation, camouflage treatment was provided as the patient was not concerned about these lesions. Additionally, these lesions are considered a valuable clinical marker for treatment response and overall survival in melanoma.

## Discussion

Cutaneous adverse drug reactions secondary to immune checkpoint inhibitors are quite common and are reported in 30-50% of the patients treated with these drugs. Individual appearance of Sweet's syndrome, vitiligo, and eczematous eruption after the initiation of immune checkpoint inhibitors has been described in the literature, but their coexistence in the same patient is relatively rare [1]. In the present case, the patient developed lesions suggestive of Sweet’s syndrome after eight months of nivolumab therapy. Vitiligo-like depigmentation and eczematous eruption were noticed two months and four months after starting nivolumab therapy, respectively. After a thorough literature review, we were unable to find Sweet's syndrome secondary to nivolumab, although there are reports secondary to other ICIs, including Ipilimumab (CTLA-4-directed monoclonal antibody), causing Sweet's syndrome [2-4].

Melanoma-associated vitiligo can occur in up to 2-25% of melanoma patients receiving ICI therapy [1]. It is considered a good clinical marker for response and overall survival in melanoma [1]. Other common cutaneous side effects of ICI, including nivolumab, are eczematous eruption, maculopapular rash, granulomatous reaction, lichenoid reaction, pruritus, and bullous pemphigoid [1].

After a detailed literature review, we were unable to find any reports of nivolumab-induced Sweet's syndrome or concomitant manifestations of vitiligo, Sweet's syndrome, and eczema in the same patient. As ICI therapy is becoming a prevalent anti-cancer treatment, awareness regarding these adverse effects is important for the correct diagnosis and management of patients.

## Conclusions

Treatment of melanoma patients with nivolumab can lead to various cutaneous side effects, including Sweet's syndrome, lichenoid eruption, eczematous eruption, maculopapular rash, and vitiligo. A clinical suspicion of Sweet’s syndrome should be considered if a patient with melanoma who is on nivolumab therapy develops fever and suggestive cutaneous lesions. Sweet's syndrome after immune checkpoint inhibitors has been mostly treated with oral corticosteroids in the literature. However, in our case, the patient was well-managed with dapsone. In literature regarding neutrophilic dermatoses secondary to these drugs, it is still not clear whether the drug should be discontinued or reactions can be managed without stopping a checkpoint inhibitor, as withdrawing this class of drugs can complicate a patient's oncologic regimen. In our patient, the drug was stopped till subsidence of lesions of sweet syndrome and then restarted at a low dose with close follow-up. The presence of vitiligo-like depigmentation in melanoma patients on anti-PD-1 therapy is a good clinical marker of treatment response, and a re-pigmentation of vitiligo suggests disease relapse. Physicians should be on a close lookout for these cutaneous adverse effects while prescribing immune checkpoint inhibitors like anti-PD-1 therapy.
